# The Effects of Embryonic Cerebrospinal Fluid on The Viability and Neuronal Differentiation of Adipose Tissue-Derived Stem Cells in Wistar Rats

**DOI:** 10.22074/cellj.2020.6560

**Published:** 2019-10-14

**Authors:** Mohammad-Hossein Mohammadi-Mahdiabadi-Hasani, Mohammad Nabiuni, Kazem Parivar, Siamak Yari, Alireza Sahebi, Jaleel Miyan

**Affiliations:** 1. Department of Cell and Molecular Biology, Faculty of Biological Sciences, Kharazmi University, Tehran, Iran; 2. Department of Biology, Sciences and Research Branch, Islamic Azad University, Tehran, Iran; 3. Department of Biology, Faculty of Sciences, Bu-Ali Sina University, Hamedan, Iran; 4. Faculty of Biology, Medicine and Health Division of Neuroscience and Experimental Psychology, The University of Manchester, Manchester, UK

**Keywords:** Adipose Tissue, Cerebrospinal Fluid, Neuronal Differentiation, Stem Cells

## Abstract

**Objective:**

The embryonic cerebrospinal fluid (e-CSF) contains various growth factors and morphogens. Recent studies showed that e-CSF plays significant roles in embryonic brain development. Adipose tissue-derived stem cells (ADSCs) have a mesodermal origin that can be differentiated into mesodermal and ectodermal lineages. This study aimed to evaluate the effects of e-CSF on the proliferation, viability, and neural differentiation of ADSCs in rats.

**Materials and Methods:**

In this experimental study, adipose tissue was dissected out from the inguinal region of adult male rats. Then, ADSCs were isolated by enzymatic digestion from adipose tissues and mesenchymal cells were confirmed using the flow cytometry analysis that measured the cell surface markers including CD90, CD44, CD73, CD105, CD34, CD45, and CD11b. The multi-potential characteristics of ADSCs were assessed by osteogenic and adipogenic potentials of these cells. Under suitable *in vitro* conditions, ADSCs were cultured in DMEM supplemented with and without additional 10% e-CSF. These fluids were collected from Wistar rats at the E17, E18, and E19 gestational ages. Cellular proliferation and viability were determined using the MTT assay. Immunocytochemistry was used to study the expression of β-III tubulin in ADSCs. The neurite outgrowth of cultured cells was assessed using the ImageJ software.

**Results:**

The results of the present study demonstrated that the viability of ADSCs in cell culture conditioned with E17 and E18 e-CSF were significantly increased in comparison with controls. Cultured cells treated with e-CSF from E18 and E19 established neuronal-like cells bearing long process, whereas no process was observed in the control groups or cultured cells treated with E17 e-CSF.

**Conclusion:**

This study showed that e-CSF has the ability to induce neuronal differentiation and viability in ADSCs. Our data support a significant role of e-CSF as a therapeutic strategy for the treatment of neurodegenerative diseases.

## Introduction

Cerebrospinal fluid (CSF) is a clear and colorless fluid, secreted mainly (about two-third of its volume) from the epithelial structure in the choroid plexus, and it could also be released from other regions in the brain such as capillaries surrounded by astrocytes, ependymal epithelium of the ventricles, and subarachnoid plexus (1). The CSF secretion starts at the early stages of the neural tube development. It contains many morphogenic and growth factors such as neurotrophin-3 (NT-3), hepatocyte growth factor (HGF), transforming growth factor-β (TGF-β), insulin-like growth factor (IGF), nerve growth factor (NGF-3), basic fibroblast growth factor (b-FGF), and brain-derived neurotrophic factor (BDNF), involved in the proliferation, differentiation, and survival of neural cells (2, 3).

Previous studies have shown that embryonic cerebrospinal fluid (e-CSF) is a rich source of proteins, which are involved in the proliferation, differentiation, and migration of neural progenitor cells during brain development. E-CSF affects the neuroepithelial cells by regulating the proliferation, differentiation, and survival of these types of cells. Similar to CSF, e-CSF is a cocktail of various growth and morphogenesis factors (4, 5).

Adult stem cells are characterized by self-renewal ability, long-time survival, and multipotency (6). Compared with the embryonic stem cells, adult stem cells are immunecompatible, non-tumorigenic, and working with them has no ethical issues (7).

Due to easy accessibility, mesenchymal stem cells (MSCs)-commonly obtained from the bone marrow - are a new cell resource for clinical practice and research (8). However, the clinical use of bone marrow-derived stem cells is restricted due to its highly invasive nature required for cell extraction and low proliferative capacity of the isolated cells (9). In a search for an alternative MSCs source, recently MSCs has been isolated from adipose tissues (10).

Adipose tissue-derived stem cells (ADSCs) have high proliferation potential that can be differentiated into a variety of mesenchymal cell lineages such as osteoblasts and adipocytes. They also have regenerative properties and potency to differentiate into nerve and Schwann cells (11, 12). As they could be obtained using minimally invasive methods and have high proliferation capacity, ADSCs are a promising tool for regenerative medicine (13). Thus, the current study aimed to evaluate whether e-CSF can induce neural proliferation and differentiation in ADSCs, as well as assessing the impact of e-CSF on the viability of ADSCs.

## Materials and Methods

### Animals

In this experimental study, 22 male and 56 pregnant female Wistar rats were used. The animals were kept in an animal house located in the Department of Biology at the Kharazmi University. They were kept in large rat boxes with free access to food and water under a 12:12 light/dark cycle. All animals were treated according to the guidelines set by the Kharazmi University based on the National Institutes of Health (NIH) Guidelines for the Care and Use of Laboratory Animals (C: 616/919). Individual male and female rats were mated and checked daily for the vaginal plug presence, designated as embryonic day 0 (E0). The embryonic age was calculated from E0. At specific times, pregnant rats were euthanized with urethane (1.5 g/kg urethane i.p., Sigma, UK), and the uterus was quickly evacuated into an icebox. Then, litters were immediately separated from pregnant Wistar rats. Each pregnant rat delivered separated from litters in each delivery.

### Collection of embryonic cerebrospinal fluid samples

To evaluate e-CSF effects on ADSCs cultures, e-CSF was collected from cisterna magna of rat embryos on day 17 (E17), 18 (E18), and 19 (E19) using a glass micropipette. Because of large fluid space, the cisterna magna is an ideal site for extracting uncontaminated e-CSF. Besides, the cisterna magna is exposed upon dissecting the skin and the removal of the overlying muscles. Due to the lack of bone formation in this area and its flexibility, the risk of blood contamination in CSF would be decreased. All samples were collected in sterile microtubes and centrifuged immediately at 4000 rpm for 5 minutes to separate the fluid from cellular debris. Afterward, the supernatant was transferred and preserved in a new sterile tube. The samples were stored at -40˚C until the subsequent use. About 10 to 50 μl of e-CSF was collected from each litter. All steps mentioned above were carried out on an ice box to avoid denaturation of e-CSF proteins (14).

### Isolation and culture of adipose tissue-derived stem cells

Adult male rats (180-220 g) were anesthetized with urethane (1.5 g/kg i.p.). Under sterile conditions, all parts of white adipose tissue samples were isolated from the abdominal inguinal region and transferred into phosphate-buffered saline (PBS, Gibco, UK). Adipose tissues were minced to pieces in sizes between 1 and 2 mm3 using scissors and then washed repeatedly with equal volumes of PBS to remove blood cells. Next, each piece was transferred into a falcon tube containing 0.075% collagenase type I enzyme (Sigma Aldrich, USA). Tubes were placed on a shaker incubator at 37˚C for 30 minutes. After that, samples were centrifuged at 2000 rpm for 5 minutes. Undigested pieces were removed, and the remaining suspension containing stem cells were collected. Extracted ADSCs were cultured in Dulbecco’s Modified Eagle Medium (DMEM, Gibco, Invitrogen, USA) containing 10% fetal bovine serum (FBS, Gibco, UK) and 1% penicillin/streptomycin. The cultures were kept in a humidified atmosphere incubator at 37˚C with 5% CO_2_. The culture media were renewed every three days (15). In this study, cell cultures were divided into five groups: I. Control: without e-CSF treatment, II. E17: treatment with e-CSF from E17 (10% v/v), III. E18: treatment with e-CSF from E18 (10% v/v), IV. E19: treatment with e-CSF from E19 (10% v/v), and V. β-Mercaptoethanol (β-ME): treatment with β-ME (10 ng/ml) as the positive control.

### Cell viability assay

In the present study, the harvested ADSCs were rinsed with PBS, transferred into 24-well plates (7×10^4^ cells/well), treated with e-CSF (E17, E18, and E19) (10% v/v), and β-ME (10 ng/ml, Sigma Aldrich, USA) for six days. The wells without any treatment were considered as controls. Cell survival and viability were measured by the MTT assay. MTT (3-(4,5-Dimethylthiazol-2 thiazolyl)-2,5- diphenyl 2 tetrazolium bromide) is a yellow tetrazolium dye that responds to activated mitochondrial dehydrogenases and changes the yellow color of samples to dark blue formazan crystals. The cells were incubated with MTT (5 mg/mL in PBS, Merck, Germany) at 37˚C for 3 hours. Finally, the absorbance was recorded at 570 nm using a plate reader (ELx808TM, BioTek® instruments, USA). Each experiment was repeated in triplicate (16). 

### Adipose tissue-derived stem cells multi-lineage differentiation potential

Another feature of mesenchymal cells is their ability to differentiate into osteocytes and adipocytes. Therefore, in the present study, the differentiation of MSCs into adipose and bone cells was evaluated. For this purpose, ADSCs were harvested at three passages and cultured in the osteogenic and adipogenic inducing medium for 21 days. Osteogenic differentiation culture media consisted of DMEM-LG (low glucose) supplemented with 10% FBS, 0.1 μM dexamethasone, 10 μM β-glycerophosphate, and 50 μM ascorbate. To assess mineralization, cultures were stained with 2% Alizarin Red (Sigma-Aldrich, USA). Adipogenic inducing culture media contained DMEM-LG, supplemented with 10% FBS, 0.5 mM IBMX (3-isobutyl-1-methylxanthine), 10 mg/ml insulin, 1 mM dexamethasone, and 100 mM indomethacin. Cells were stained with Oil Red (Sigma-Aldrich, USA) for the detection of adipocytes (17).

### Flow cytometry analysis

After second consecutive passages, to measure the cell surface markers of MSCs and to confirm their development, we used the following conjugated antibodies: PE Mouse Anti-Rat CD44, PE Mouse Anti- Rat CD73, PE Mouse Anti-Rat CD105, FITC Mouse Anti-Rat CD90, PE Mouse Anti-Rat CD34, PE Mouse Anti-Rat CD45, PE Mouse Anti-Rat CD11b, and isotype control antibody. After trypsinization of the cells with 0.25% trypsin/ ethylenediaminetetraacetic acid (EDTA) solution, they were re-suspended in PBS and counted using hemocytometer. A number of 1×106 cells were incubated in fluorochrome-conjugated antibody at a dilution ratio of 1:10 at room temperature for 20 minutes in the dark place. The stained ADSCs were analyzed using a BD FACSCaliber™ flow cytometer (BD Biosciences, USA) and the FlowJo software (version 10.4). A total of 200,000 cells were assessed in each sample.

### Morphological properties and neurite-like processes assessment

In order to evaluate the morphology and growth rate of ADSCs, cells were imaged using an inverted microscope (Olympus, Japan) and the obtained images were analyzed by the ImageJ software (NIH). The morphological properties of the cells, as well as the length of neurite-like processes, were compared among cell treatments.

### Immunocytochemistry

In this study, β-III tubulin was considered a neural differentiation marker. After three times washing with PBS, cells were fixed with cold 4% paraformaldehyde for 15 minutes. Then, 0.1% Triton X-100 (Merck, Germany) was employed for the increase of cell permeability at room temperature for 30 minutes. The cells were blocked with 1% BSA (Sigma-Aldrich, USA) in T-PBS (Tween 20 in PBS) (T-PBS, Gibco, UK) at room temperature for 1 hour. After that, the cells were incubated at 4˚C overnight in the presence of anti-β III tubulin (1:100 Dilution, Abcam, UK) as a primary antibody. The next day, cells were rinsed three times with T-PBS and incubated with a Cy3-conjugated secondary antibody (1:300 Dilution, Abcam, UK) at room temperature for 1 hour. Finally, cells were washed with PBS, and nuclear staining was carried out with 4’,6-Diamidino-2-Phenylindole (DAPI, Sigma- Aldrich, USA). Photomicrography was done under a fluorescent microscope (Olympus, Japan).

### Statistical analyses

Ordinary one-way analysis of variance (one-way ANOVA) was applied for the statistical analysis, followed by Tukey’s post hoc test to compare multiple groups. Data are expressed as the mean and standard error of the mean (mean ± SEM). The P<0.05 was considered statistically significant. Data were analyzed using the the IBM SPSS Statistics for Windows, Version 24.0. Armonk, NY: IBM Corp. (Released 2016).

## Results

### Morphological characteristics and pluripotency of adipose tissue-derived stem cells

**Fig 1:**
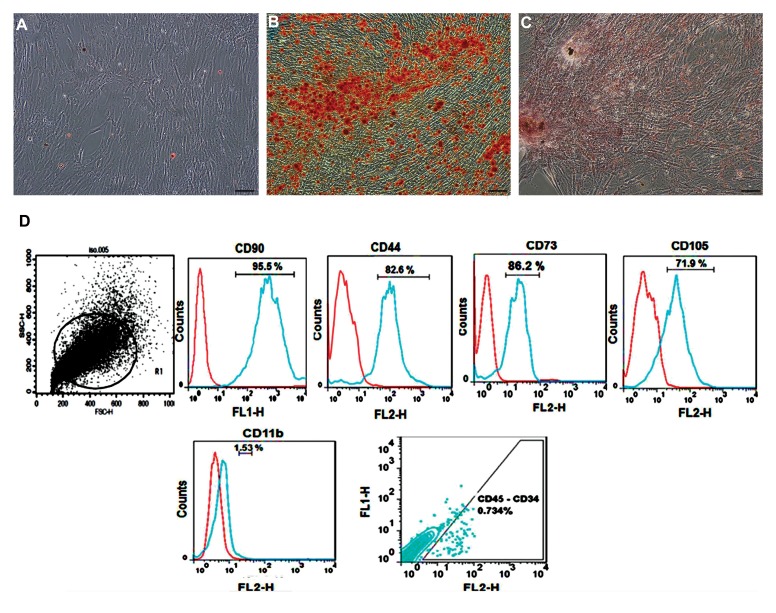
Morphological characteristics and pluripotency of adipose tissue-derived stem cells (ADSCs). Photomicrographs of ADSCs show typical spindle-shaped morphology. A. Cultured ADSCs in complete growth medium (control group), B. Adipogenic differentiation of ADSCs (Oil Red staining), C. Osteogenic differentiation of ADSCs (Alizarin Red staining), (scale bars: 100 μm), and D. Flow cytometry histograms indicating immunophenotype of mesenchymal stem cells isolated from rat’s adipose tissue. The cells expressed the cell surface markers namely CD90, CD44, CD73, and CD105, but not CD34, CD45, and CD11b.

Cultured cells were adherent and had spindle-shaped morphology. Besides, cultured ADSCs were pluripotent and differentiated into osteoblasts and adipocytes in specific conditioned media. Alizarin red and Oil Red staining were carried out for the confirmation of ADSCs differentiation into osteoblasts and adipocytes (Fig .1A-C).

### Flow cytometry analysis of the expression of stem cells markers in adipose tissue-derived stem cells population

Cell surface markers were utilized on the second passage to characterize ADSCs population. The flow cytometry analysis of cell surface markers showed that these cells expressed CD90, CD44, CD73, and CD105 as MSCs markers, whereas they did not express CD34, CD45, and CD11b markers (Fig .1D).

### Effects of embryonic cerebrospinal fluid on adipose tissue-derived stem cells viability

**Fig 2:**
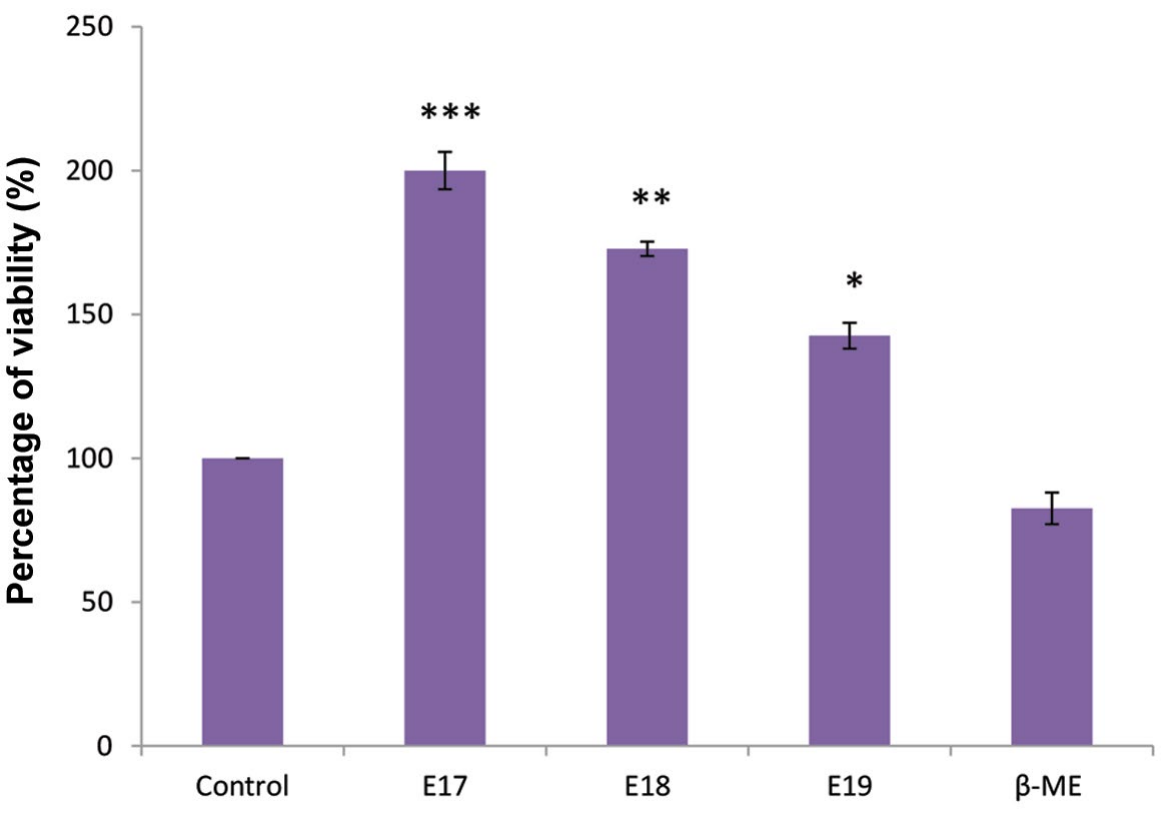
Survival rates of adipose tissue-derived stem cells (ADSCs) in cell culture treated with embryonic cerebrospinal fluid (e-CSF, 10% v/v) and β-Mercaptoethanol (β-ME, 10 ng/ml) using the MTT assay (n=5). All cultured ADSCs indicated an increase in viability after treatment with e-CSF. The increase was significant in treatment with CSF from embryonic days 17, 18 and 19 (E17, E18, and E19). Data are expressed as the percentage of control levels (cell culture without the addition of CSF). *; P<0.05, **; P<0.01, and ***; P<0.001 as compared with the control group.

After six days of treatment, viability assessment was implemented in all cultured groups. As shown in Figure 2, the MTT absorbance was increased significantly in cultured cells conditioned with CSF (10% v/v) from E17, E18, and E19. However, the MTT absorbance was decreased after the cells were treated with β-ME (positive control) compared with the control group. The highest viability was observed at E17 and E18 in e-CSF treatment (Fig .2).

### The Effects of embryonic erebrospinal fluid on neurite outgrowth of adipose tissue-derived stem cells

**Fig 3:**
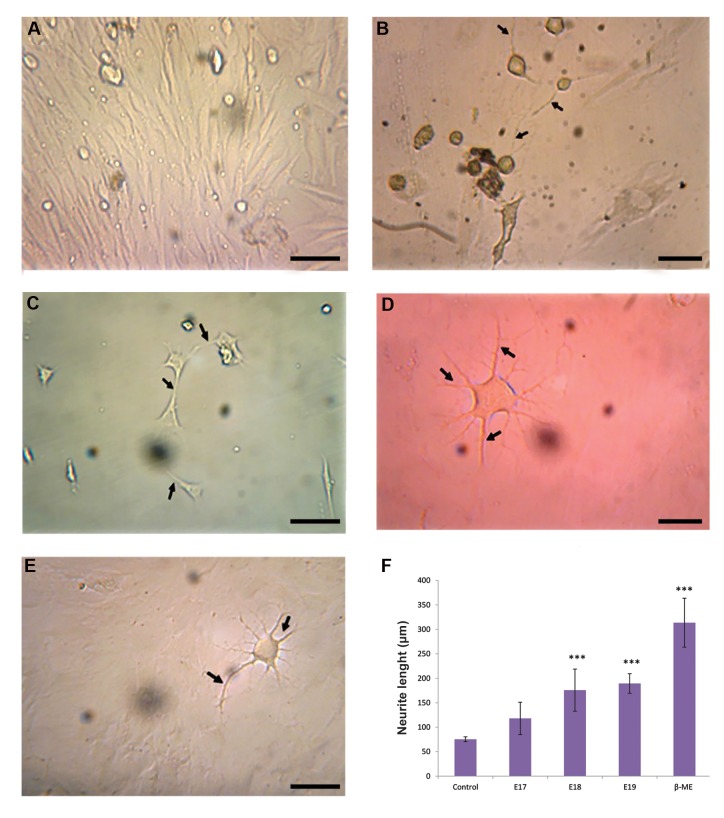
Morphological differentiation and neurite outgrowth length of adipose tissue-derived stem cells (ADSCs) after a 6-days period treatment with embryonic cerebrospinal fluid (e-CSF, 10%v/v) and β-Mercaptoethanol (β-ME, 10 ng/ml). **A. **Control without treatment, **B.** Treatment with β-ME, **C.** Treatment with e-CSF at E17, D. Treatment with e-CSF at E18, E. Treatment with e-CSF at E19. Treatment of ADSCs with β-ME and e-CSF at E17, E18, and E19 showed neurite outgrowth (arrows) and morphological differentiation as compared to the control group. No morphological changes were observed in the control group (scale bar: 10 μm), and F. The length of neurites in ADSCs treated with e-CSF at E18 and E19 and β-ME (positive control) was significantly increased compared to the control sample. Data are presented as the mean ± S.E.M. ***; P<0.001

Photomicrographs of the cultured cells are shown in Figure 3. Neurite outgrowth and morphological differentiation were observed in treatment groups with β-M (Fig .3B) and e-CSF at E17 (Fig .3C), E18 (Fig .3D), and E19 (Fig .3E), whereas no morphological differentiation was evident in the control group (Fig .3A). The diameter of ADSCs was measured by the ImageJ software. ADSCs treatment with e-CSF at E18 and E19 significantly increased neurite outgrowth compared with the control samples, but the increase in the experimental group treated with e-CSF at E17 was not statistically significant (Fig .3F).

### Immunocytochemistry characteristics of differentiated adipose tissue-derived stem cells

**Fig 4:**
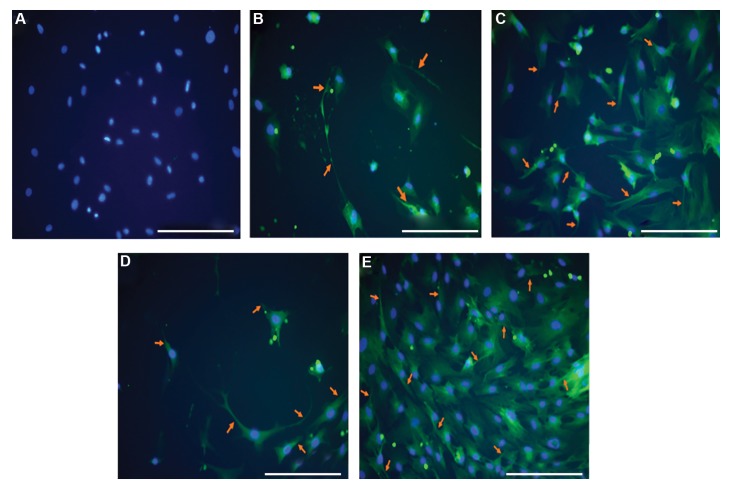
Immunofluorescence localization of β III-tubulin (orange arrow) in adipose tissue-derived stem cells (ADSCs) (green) treated with embryonic cerebrospinal fluid (e-CSF) at E17, E18, and E19 (10% v/v) and β-Mercaptoethanol (β-ME, 10 ng/ml). **A.** ADSCs in the control group without e-CSF expressed no β III-tubulin, **B.** ADSCs treated with β-ME, **C.** ADSCs treated with e-CSF at E17, **D.** E18, and **E.** E19. Cell nuclei are stained with DAPI (blue) (scale bars: 100 μm).

For neural induction, ADSCs were incubated whit β-ME (traditional neural inducer) as the positive control. As shown in Figure 4, the majority of ADSCs in this condition have a high level of β III-tubulin expression. Moreover, the number of cells were positive for β III-tubulin were significantly increased in cultured cells conditioned with CSF at E17, E18, and E19. However, no changes were observed in the expression of β III-tubulin in ADSCs of the control group (Fig .4).

## Discussion

Considering the benefits of the ADSCs in comparison with other sources of MSCs, e.g. being less invasive compared to lipo-aspiration, having less ethical issues (10, 18), providing more homogenous stem cells with less variations in morphological features, and being ideal for the assessment of environmental changes (19); in the present study, ADSCs were taken into account as an appropriate model for our investigation.

Our flow cytometry data showed that CD90, CD44, CD73, and CD105 as MSCs-specific markers were expressed by ADSCs, whereas CD34, CD45, and CD11b were not, demonstrating the mesenchymal origin of ADSCs. ADSCs also share some morphological characteristics with MSCs, such as being spindle-shaped and possessing fibroblast-like properties (10, 20). MSCs are highly multipotent and can differentiate into mesodermal lineage such as adipogenic, chondrogenic, and osteogenic cells (17). Our findings confirmed that ADSCs could differentiate into bone and adipose tissues. There are several studies that applied different approaches for the neural induction of MSCs, such as β-Mercaptoethanol, valproic acid, butylated hydroxyanisole, hydrocortisone, azacytidine, isobutylmethylxanthine, indomethacin, insulin (17, 19, 21), glial growth factors, as well as a mixture of bFGF, platelet-derived growth factor, BDNF, and retinoic acid (22). Briefly, the protocols mentioned earlier could be divided into two main groups: i. Chemical and ii. Growth factor-based methods. It has been demonstrated that chemical-based induction methods lead to the production of nonfunctional neuron-like cells and induce an increment in the rate of apoptosis (23).

The CSF contains various biological factors such as neuropeptides and neurotransmitter, possessing different concentrations during various gestational ages. Several studies also showed that adult CSF isolated from human and rat could stimulate the proliferation and viability of neural stem cells.

Additionally, e-CSF comprises of several growth factors including TGF-β, NGF, BDNF, NT-3, and IGF (24, 25). In recent years, several studies demonstrated that the changes in the levels of CSF factors have multiple impacts on the proliferation and differentiation of brain cells in different animal models under various conditions (24). In the present study, we observed that the application of e-CSF, as a growth factor-based method, induced morphological changes in the neural phenotype of MSCs and increased cell viability. Yari et al. (26) also indicated that e-CSF enhanced the proliferation of neural progenitor cells and increased neurosphere size in culture media. Mercaptoethanol, as a chemical inducer, could also cause ADSCs differentiation in culture media, but significantly reduced cell viability (27).

Dual effects of e-CSF on ADSCs proliferation and differentiation are probably caused by various growth factors, their concentration changes in different embryonic stages, and their interaction in each step. Several reports demonstrated that e-CSF collected from different embryonic days of the rat brain (E16, E17, E18, E19, and E20) has different effects on neuronal progenitors derived from the embryonic brain of rats. By E16 and E18, these effects have stimulatory roles on the proliferation and survival of neuronal progenitors. It has been implicated that e-CSF extracted at E20 and E19 has a significant impact on the differentiation (26, 28, 29). Nabiuni et al. (28) evaluated the effect of rat embryonic CSF on PC12 cells. They observed that the proliferation and viability of PC12 cells that underwent exposure to CSF at E18 are significantly elevated, but PC12 cells cultured in media supplemented with b-FGF (neural inducer) and CSF at E20, represented neurallike morphology. In another study, Yari et al. (26) investigated the effect of embryonic CSF on neural progenitors cells. In this study, e-CSF extracted at E18 induced the proliferative impact on neural progenitors cells and significantly increased cell viability. In the presence of e-CSF at E18 and E19, the neuronal process growth in cultured ADSCs was also markedly increased compared with the control samples.

Previous studies indicated that e-CSF isolated at E18 and E19 might differentiate cells into neuronal cells (26, 28). The results of the neuronal process growth confirmed the differentiation of these cells towards neurons. In fact, the effects of e-CSF extracted from different gestational days (E17, E18, and E19) on cultured ADSCs are age-dependent and probably due to the changes in the concentration of various growth factors in a timedependent manner.

The mechanisms by which the e-CSF contents are altered in different time points is probably related to the developmental changes in the CSF-brain barrier. It has been reported that the permeability of CSF-brain barrier changes in an age-dependent manner during embryonic and adult life. The changes in permeability are due to the differential distribution of junctional proteins in the CSFbrain barrier. Therefore, alterations in permeability could be a source of e-CSF composition changes in different gestational periods (30).

In this study, there were limitations on e-CSF extraction from the cisterna magna region of rats’ embryo. Due to the low volume of e-CSF obtained from this area, we suggest the extraction of e-CSF could be performed on animal models since they provide a high amount of e-CSF for the experiment. Also, we propose the application of other techniques such as real-time PCR for the precise evaluation of the expression of genes involved in differentiation and proliferation of ADSCs exposed to e-CSF.

## Conclusion

Previous studies have shown that various factors with different concentrations are presented in embryonic CSF and their level varies on different embryonic days. The results of this study indicated the changes in the e-CSF compositions in a time-dependent manner, which had a positive impact on ADSCs survival and differentiation. Considering specific properties of ADSCs, their differentiation in response to exposure to e-CSF may be regarded as a novel therapeutic strategy for the treatment of neurodegenerative disorders.
